# Geometry-Driven Polarity in Motile Amoeboid Cells

**DOI:** 10.1371/journal.pone.0113382

**Published:** 2014-12-10

**Authors:** Oliver Nagel, Can Guven, Matthias Theves, Meghan Driscoll, Wolfgang Losert, Carsten Beta

**Affiliations:** 1 Institute of Physics und Astronomy, University of Potsdam, Potsdam, Germany; 2 Department of Physics, University of Maryland, College Park, Maryland, United States of America; University of Heidelberg Medical School, Germany

## Abstract

Motile eukaryotic cells, such as leukocytes, cancer cells, and amoeba, typically move inside the narrow interstitial spacings of tissue or soil. While most of our knowledge of actin-driven eukaryotic motility was obtained from cells that move on planar open surfaces, recent work has demonstrated that confinement can lead to strongly altered motile behavior. Here, we report experimental evidence that motile amoeboid cells undergo a spontaneous symmetry breaking in confined interstitial spaces. Inside narrow channels, the cells switch to a highly persistent, unidirectional mode of motion, moving at a constant speed along the channel. They remain in contact with the two opposing channel side walls and alternate protrusions of their leading edge near each wall. Their actin cytoskeleton exhibits a characteristic arrangement that is dominated by dense, stationary actin foci at the side walls, in conjunction with less dense dynamic regions at the leading edge. Our experimental findings can be explained based on an excitable network model that accounts for the confinement-induced symmetry breaking and correctly recovers the spatio-temporal pattern of protrusions at the leading edge. Since motile cells typically live in the narrow interstitial spacings of tissue or soil, we expect that the geometry-driven polarity we report here plays an important role for movement of cells in their natural environment.

## Introduction

Many essential biological processes rely on the ability of eukaryotic cells to move [1]. Prominent examples are embryonic development, immune responses, and the spreading of metastatic cancer cells. The forces that drive these types of locomotion are provided by the actin cytoskeleton [2], [3]. In particular, during amoeboid motion, protrusions are formed at the leading edge by actin polymerization against the cell membrane, while the contractile action of myosin II retracts the rear of the cell body. The asymmetric distribution of cytoskeletal components and their associated forces are hallmarks of cell polarity, typically associated with an asymmetric morphology and a polar distribution of other subcellular components like signaling proteins and membrane lipids [4]. Symmetry breaking into a polar configuration may occur spontaneously or in response to external cues. For example, during eukaryotic gradient sensing, cells detect concentration differences of chemoattractants across their cell body and respond by asymmetrical redistribution of intracellular signaling components. The asymmetric signaling pattern triggers a polar rearrangement of the actin cortex that results in directed chemotactic movement towards the source of the chemoattractant [5].

To date, our understanding of actin-driven motility and cell polarity mostly depends on studies of cells on planar open substrates. In these migration assays the leading edge protrusion and back retraction were investigated in detail, including the associated cortical dynamics, the role of substrate adhesion, and traction forces [6]. Statistical analyses of cell trajectories complement our understanding of cell locomotion on open surfaces, see e.g., Refs. [7]–[10]. However, the native environment of a motile cell is very different from the artificial laboratory setting of a planar surface. For example, differentiating cells in an embryo or leukocytes that are leaving the blood vessel to reach a site of injury have to squeeze through the surrounding tissue. How do cells move through such confined three-dimensional environments to fulfill their designated tasks?

In recent years, interstitial motility has attracted growing attention in the field of motility research. It was shown that cell motion in confined three-dimensional matrices differs significantly from the behavior on a two-dimensional planar surface. For example, motion in a confined three-dimensional environment is more similar to one-dimensional rather than two dimensional motion [11], [12]. Cancer cells may show directed and persistent movement when confined to narrow channels [13], [14]. Also, functional integrins that play an important role during leukocyte migration on planar substrates were found to be dispensable for movement in a confined three-dimensional environment [15] — a result that has also stimulated theoretical descriptions of dendritic motility [16]. Additionally, the arrangement of the actin network at the leading edge is altered during interstitial migration [17] and cells may switch between different types of protrusion to adapt their mechanism of locomotion to the confined environment [12], [18]. Recently, also hydraulic pressure was identified as a physical cue that may bias cell migration in narrow channels [19]. Even a novel water permeation-based propulsion mechanism has been identified that drives tumor cell migration in confined environments independently of the actin cytoskeleton [20]. Despite these insights, our knowledge of interstitial motility remains sparse.

In this study, we investigate how confinement influences the polarity of motile amoeboid cells. We observe that inside narrow microchannels, cells of the social amoeba *Dictyostelium discoideum* may spontaneously switch into a state of highly persistent unidirectional motion. No chemical gradients are required to induce this polar behavior. We define polarity based on the strongly asymmetric, unidirectional mode of movement. To distinguish this notion of polarity from definitions based on the localization of specific intracellular polarity markers, we call these cells mechanically polarized. During the persistent movement, the actin cortex exhibits a dynamic leading edge, where protrusions rapidly form and travel across the cell front in a left-to-right zigzag fashion. At sites of contact with the microchannel walls, dense actin-rich zones are observed that remain stationary with respect to the walls, while cells move through the channel. We observed enrichment of myosin II at the back of the cell. However, experiments with knockout mutants show that myosin II is not required for this type of persistent motion. We rationalize our findings in the framework of a biased excitable network model with an additional putative polarity marker. The model explains the geometry-induced persistent cell motion and its responses to external perturbations.

## Materials and Methods

### Cell culture

*D. discoideum* AX2 wild-type cells were cultivated in HL5 medium (Formedium, Norwich, England) at 22°C on polystyrene Petri dishes (Primaria, Falcon, BD Becton Dickinson Europe, France), or shaken in suspension at 150 rpm. For fluorescence imaging of actin and myosin II dynamics, we used *Dictyostelium* cell lines coexpressing LimE-RFP and myosin II-GFP in an AX2 background and LimE-GFP in myosin II heavy-chain null (HS2205), cultured with selection markers blasticidin and/or G418. Before the experiments, the cells were washed and transferred into 25 mL shaking phosphate buffer solution (150 rpm), which consists of 14.6 mM KH_2_PO_4_ and 2 mM Na_2_HPO_4_ (Merck KGaA, Darmstadt, Germany). The resulting solution has a pH-value of 6.0. In this solution the cell were starved. After approximately three hours, the cells were centrifuged at 1000 rpm for 3 min and filled into the microfuidic channel. The cells were left for 30 min inside the channel to attach to the glass coverslip before the experiment was conducted. During the experiment, a gentle fluid flow was applied to the microchannels with a syringe pump to provide fresh buffer and oxygen to the cells.

### Microfluidics

The microfluidic devices were fabricated by standard soft lithography [21] (a detailed layout of the microchannel network is shown in the Figure S5). A silicon wafer was coated with a 20 µm photoresist layer (SU-8 50, Micro Resist Technology GmbH, Berlin, Germany) and patterned by photolighography to obtain a microstructured master wafer. Polydimethylsiloxane (PDMS, 10∶1 mixture with curing agent, Sylgard 184, Dow Corning GmbH, Wiesbaden, Germany) was poured onto the wafer and cured for 2–3 h at 75°C. To produce the microfluidic device, a PDMS block containing the microchannels was cut out, and inlets were punched through the PDMS with the help of a syringe needle. Afterwards, a glass coverslip (24 × 40 mm, Menzel Gläser) was sealed to the PDMS block following a 2–3 min treatment in air plasma (PDC 002, Harrick Plasma, Ithaca, USA) to close the microchannels.

### Image acquisition and analysis

We imaged cells with both bright field and confocal fluorescence microscopy for two hours with frame rates of 0.1/sec. and 0.5/sec., respectively. Cell trajectories were computed from both types of imaging data. In the case of bright field images, we determined the cell trajectories using the Manual Tracking plugin of ImageJ (National Institutes of Health, USA). In the case of fluorescence images, we used a custom-made MATLAB-based next neighbor particle tracking algorithm (MathWorks, Ismaning, Germany). We obtained velocities from the cell trajectories by calculating the displacement of the cell centroid between consecutive frames.

For cell shape analysis we used the active contour algorithm described in Refs. [22], [23]. This algorithm parametrized the cell boundary in each frame with 400 points. The movement of these points was tracked from one frame to the next using a least squares mapping. Based on the parametrized cell boundary, we calculated the temporal evolution of the cell shape, the local motion of the boundary points, and the distribution of F-actin and myosin II along the cell border (see the Supporting Information for details).

### Model simulations

Numerical simulations of the coupled reaction-diffusion equations for the excitable system and the polarity module were performed with a custom-written Matlab program on a one-dimensional domain of length space units with an equidistant grid of d*x* = 1 and periodic boundary conditions. An explicit Euler scheme with a fixed time step of d*t* = 0.1 sec. was employed for integration in time, and the Laplacian operator was discretized using a second-order finite-difference scheme. Simulations were performed with a duration of at least 5000 sec. For model equations and parameters, see the Supporting Information.

## Results

### Cells in narrow channels spontaneously switch to a persistent, unidirectional mode of motion

**Figure 1:**
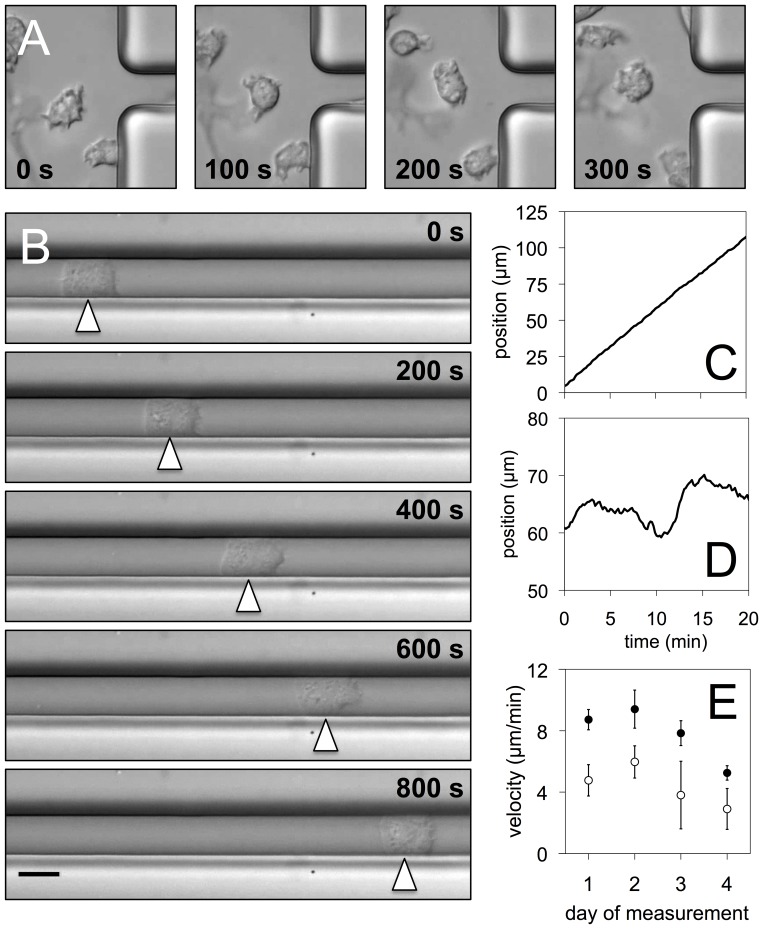
Movement in narrow microchannels — random and persistent walkers. (A) Snapshots of cells moving outside the microchannels on a planar open surface. (B) Persistent walker moving inside a microchannel, scale bar 10 µm. (C) Position of the persistent walker as a function of time. (D) Position of a random walker inside a microchannel as a function of time. (E) Average velocities of persistent (filled) and random walkers (open circles) for different experiments. The numbers of persistent and random walkers were (1) 5 and 4, (2) 5 and 3, (3) 3 and 3, (4) 10 and 5.

Using bright field microscopy, we record the motion of adherent *Dictyostelium* cells in microfluidic devices, composed of narrow channels (10 µm wide and 20 µm high) that are connected by wider inlet regions (see the Figure S5 for the layout of the device). In the inlet regions, cells can be observed that do not touch any of the channel side walls and are freely moving on a planar open surface, see Figure 1A and the corresponding movie in the Supporting Information. These cells do not show a well-defined leading edge and perform an irregular random walk. Inside the microchannels, cells from the same population split into two subgroups with clearly distinct motile behavior. The majority of the cells move randomly inside the channel, displaying frequent changes in the direction of motion and a strongly varying cell shape. Movement of these cells is interrupted by frequent pauses and results in only a small net displacement, similar to the cells outside the channels. In contrast, a second subpopulation of about one quarter of the cells moves persistently at a constant high speed along the channel without changing their direction of motion, see Figure 1B for an example. In most cases, cells of this subpopulation maintain persistent motion at uniform speed throughout the entire duration of the recording, often for more than half an hour. We refer to the first subpopulation as the “random walkers” and designate the second subpopulation as “persistent walkers”. A systematic analysis based on several of our recordings revealed that about 28% of the total population (18 out of 64 cells) moved as persistent walkers through the microchannel.

In Figure 1B, snapshots of a persistently moving cell are displayed at successive points in time that are 200 seconds apart. The corresponding movie along with further examples of persistent walkers is provided in the Supporting Information. The displacement of the persistent walker as a function of time is shown in Figure 1C and demonstrates that these cells persistently move with a constant speed along the microchannel. The displacement of a random walker can be seen in Figure 1D for comparison. It clearly contrasts with the uniform motion of the persistent walker and is characterized by frequent changes in the direction of motion. Although the cell speed shows variations between different experiments, persistent walkers always move about twice as fast as the random walkers (Figure 1E).

We have performed similar experiments with narrower and wider microchannels (5 µm and 20 µm, respectively). No persistent unidirectional motion of cells was observed in these cases. Note that we apply a small fluid flow through the channels to ensure a constant composition of the surrounding medium and to provide oxygen to the cells. Among the persistently moving cells, the number of cells that move in flow direction is roughly equal to the number of cells that move against the flow. We thus conclude that the fluid flow does not bias the directional motion of the persistent walkers.

### Persistently moving cells advance their front in a left/right alternating fashion

**Figure 2:**
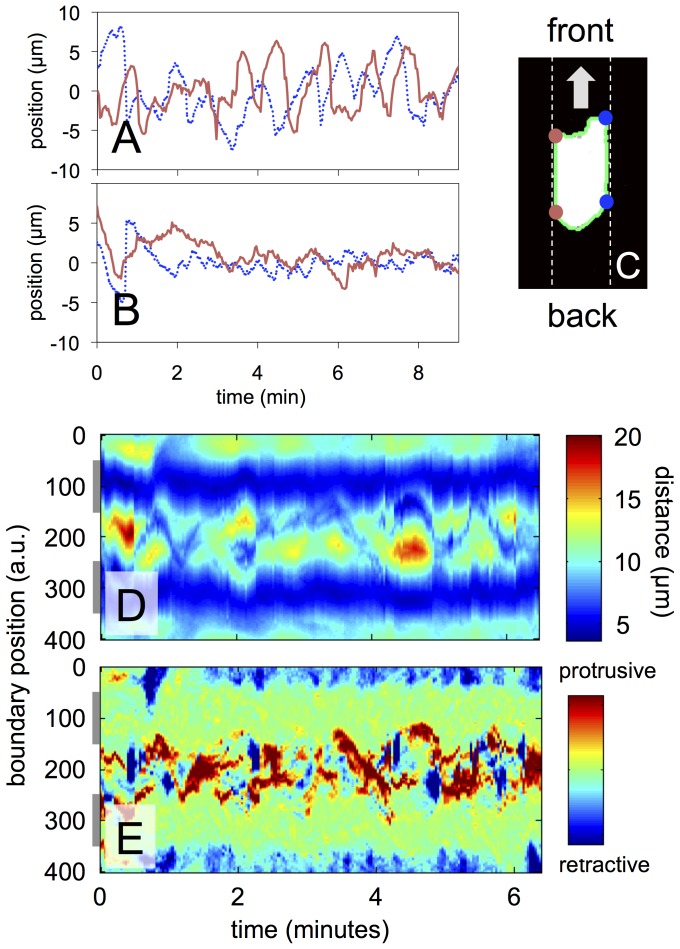
Analysis of cell shape. Position of (A) front corners and (B) back corners of a persistent walker in the co-moving frame of reference. (C) Schematic representation of a persistent walker with corners marked. (D) Kymograph of the cell shape showing the time evolution of the distance of each boundary point from the centroid of the cell. (E) Kymograph of the local motion displaying the displacement of each boundary point with respect to its previous position. Gray bars to the left of the kymographs indicate the average positions of the channel walls. — single cell example out of more than 10 cells.

The shapes of persistent walkers and random walkers are clearly distinct. During their motion, persistent walkers typically spread across the entire width of the channel and retain contact with both side walls, resulting in a characteristic rectangular shape (a representative example is shown in Figure 1B). When moving forward, the front of a persistent walker advances its left and right corners in an alternating zigzag fashion, while both corners at the back of the cell move forward synchronously. This is illustrated in Figures 2A and B, where the positions of the front and back corners are displayed in the co-moving frame (see Figure 2C for a definition of the corner positions). A quantitative analysis of the correlations between the periodically advancing front corners confirms this observation, see Figure S6.

The alternating motion of the corners at the front and the simultaneous retraction of the corners at the back of the cell is also visualized in the time evolution of the cell shape, parametrized by a constant number of equidistant points as explained in Materials and Methods. In Figure 2D, a kymograph is displayed, where the distance to the centroid of the cell is color coded for each point along the cell perimeter (boundary position). For the elongated shape of the persistent walkers, those parts of the cell border that are in contact with the channel walls are closest to the centroid (shown in blue). The alternating left/right pattern of protrusions at the leading edge corresponds to yellow and red regions, which have the largest distance to the cell centroid. All other points at the front and the back of the cell are roughly equidistant from the cell centroid and appear in green. To highlight the local protrusions and retractions more accurately, we calculated an additional kymograph, where color at each of the equidistant boundary points represents the distance the corresponding point of the boundary moves from image to image. This local motion kymograph is shown in Figure 2E, with protrusions labeled in red and retractions indicated in blue. In this representation, the channel walls are clearly visible as green (neither protruding nor retracting). At the front of the cell, we see diagonal red structures. These are signatures of protrusions that travel at constant speed in a wave-like fashion from one side of the leading edge to the other, similar to waves of protrusions seen on flat surfaces [22].

### Persistently moving cells show a distinct organization of their actin cytoskeleton

We performed fluorescence imaging experiments to study the organization and dynamics of the acto-myosin system in the persistently moving cells. We use a *Dictyostelium* cell line that co-expresses two fluorescent fusion proteins for *in vivo* imaging of actin and myosin II localization. Actin dynamics is visualized by expression of DdLimEΔcoil-RFP (LimE-RFP), an RFP-tagged Lim-domain protein with truncated coiled-coil domain that colocalizes with F-actin and has become a widely used marker to image actin dynamics in *Dictyostelium* [24], [25]. To simultaneously observe myosin II localization, we co-expressed myosin II-GFP together with LimE-RFP. Images were taken with a laser-scanning confocal microscope in an imaging plane passing through the center of the cell body.

**Figure 3:**
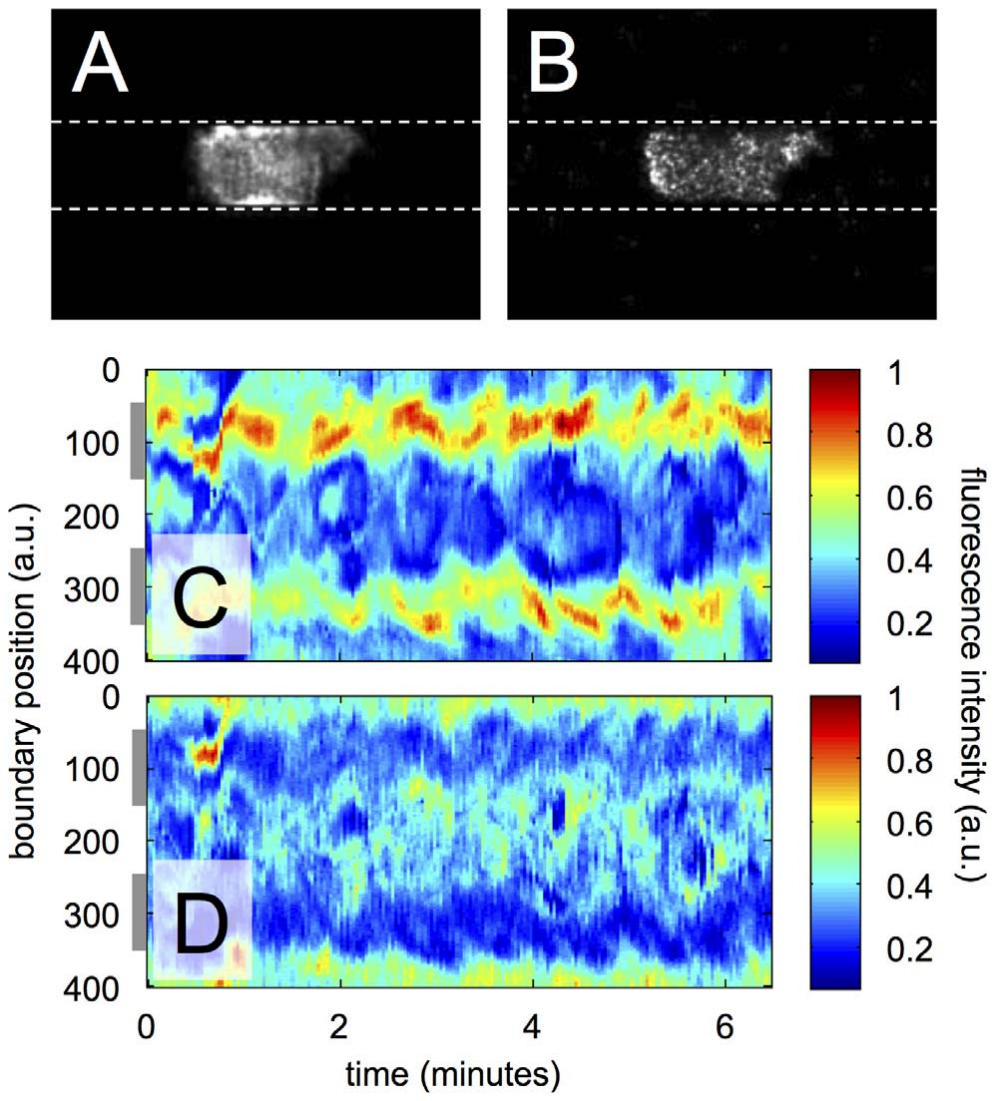
Localization of F-actin and myosin II in a persistant walker. (A) Fluorescence image showing the distribution of LimE-RFP, a marker for filamentous actin, inside a persistent walker. (B) Fluorescence image displaying the distribution of myosin II-GFP inside the same cell. Images in (A) and (B) were recorded by confocal microscopy, the cell is moving from left to right along the microchannel. Kymographs of (C) LimE-RFP distribution and (D) myosin II-GFP distribution along the cell border. Gray bars to the left of the kymographs indicate the average positions of the channel walls. — single cell example out of more than 10 cells.

In Figures 3A and B, snapshots of the actin and myosin II distributions in a persistent walker are displayed. We observe increased concentrations of F-actin at the sides of the cell that are in contact with the microchannel walls. In contrast, myosin II was mostly found at the back of the cell, where the cell body retracts. The time evolution of the cortical acto-myosin distribution is summarized in the kymographs displayed in Figures 3C and D (see Supporting Information for the corresponding movies). In Figure 3 C, the LimE-RFP fluorescence intensity is shown for each point on the cell boundary over time. The kymograph shows patches of increased actin concentration that persist at the wall-attached sides of the cell throughout the recording. To a lesser extent, F-actin also localizes at sites of protrusion formation at the cell front. A similar kymograph for the myosin II distribution is shown in Figure 3D. Here, we observe that myosin II is depleted from the actin rich areas at the wall-attached sides. At the cell front, moderate myosin II concentrations are found in places where protrusions retracted, while maximal accumulation of myosin II is observed at the back (around boundary positions 0 and 400). Additional experiments with myosin II knockout mutants demonstrate that myosin II is not essential for persistent unidirectional motion along the microchannels. Also among the myosin II deficient cells a subpopulation of persistent walkers is found similar to the wild-type (data shown in Figure S2).

To confirm that the overall acto-myosin distribution has been accurately inferred from the two-dimensional confocal slices, we performed full three-dimensional recordings of the acto-myosin distribution with spinning disk confocal microscopy (see the Supporting Information for a movie with three-dimensional rendering of a persistent walker inside the microchannel). Observations in 3D are consistent with the 2D findings reported above.

### The wall-attached cortex displays stationary actin foci

**Figure 4:**
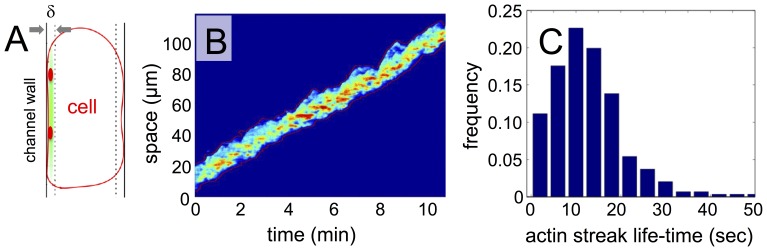
Stationary actin foci in the wall-attached cortex of persistent walkers. (A) Schematic showing the definition of the wall attached cortex with actin foci (red). (B) Kymograph displaying the fluorescence intensity in the wall-attached cortex as a function of time as the cell moves along the microchannel. (C) Histogram of the life time of actin foci in the wall-attached cortex, emerging as horizontal streaks in the kymograph in (B).

Careful analysis of the fluorescence recordings reveals that actin patches in the wall-attached cortex often remain at a stationary position with respect to the microchannel wall, while the cell moves along (see also the actin movie in the Supporting Information). The diagonal shape of the actin rich areas in the kymograph displayed in Figure 3C shows that actin foci do not move as fast as the cell itself. To establish whether the foci are stationary, we have analyzed a narrow slice of the actin cortex adjacent to the microchannel wall as indicated in Figure 4A. The kymograph in Figure 4B shows the time evolution of the LimE-RFP fluorescence intensity within this narrow region. In this case, the space-time information is displayed in the laboratory frame of reference, so that the persistent motion of the cell at uniform speed results in a diagonal structure in the kymograph. Actin structures that remain stationary with respect to the microchannel wall emerge as horizontal features in this diagram. The horizontal extent of these structures reflects their lifetime.

We can distinguish localized, horizontal streaks containing increased F-actin concentration in the space-time diagram in Figure 4B. They are a clear signature of localized actin foci that remain stationary with respect to the microchannel wall, while the cell is moving through the channel. Figure 4C shows a distribution of the lifetimes of these actin foci, accumulated from an analysis of 10 recordings of this type (see Supporting Information and Figure S1 for details). The average lifetime of actin foci is approximately 10 seconds.

## Discussion

Our observations highlight that in confined spaces, *Dictyostelium* cells can spontaneously enter a state of strong mechanical polarity that is characterized by a highly persistent unidirectional motion. Note that contrary to previous studies of *Dictyostelium* migration in microchannels [26], we did not impose any chemotactic gradient to guide the motion of cells. We observed that inside narrow microfluidic channels, a subpopulation of cells display persistent unidirectional movement along the microchannel, while the remaining part of the population performs a random walk with frequent changes in direction. Mostly, the persistently moving cells remain in contact with both side walls of the microchannel, adopting a characteristic squared shape. They however do not occlude the entire channel cross section.

### Cytoskeletal dynamics

In the cortex of the persistent walkers, most filamentous actin is concentrated in dense, F-actin rich regions at the wall-attached sides of the cell. At the leading edge, the actin cortex appears less dense and protrusions form in a highly dynamic fashion, traveling across the front membrane and extending forward. A similar cortical arrangement has been recently observed in neutrophils during chemotactic migration through narrow channels [17], where the authors suggest that a dense adherent actin network grows inwards from the contact regions with the side walls, constricting the space for a loose network at the leading edge that exhibits rapid turnover and arp2/3 dependent nucleation at the front membrane. FRAP experiments indicate that the adherent network remains stationary with respect to the channel wall [17], which is in line with our observations of stationary actin patches in the wall-attached cortex reported in Figure 4.

Myosin II was found to localize at the back of the persistently moving *Dictyostelium* cells, similar to cells that move on open surfaces. Earlier data from T-cells and leukocytes show that myosin II is not essential for interstitial migration, except for the passage through narrow gaps, where squeezing of the nucleus is required [15], [27]. It was also reported that interstitial tumor cell migration does not depend on myosin II [28]. This is in agreement with our result that myosin II-deficient *Dictyostelium* mutants undergo persistent, unidirectional motion along a narrow microchannel, similar to wild-type cells.

Taken together, our results show that amoeboid cells may switch to a state of highly persistent unidirectional motion when confined in a microchannel that corresponds in width to their own size. To elucidate the underlying mechanisms that lead to this behavior, we performed fluorescence imaging experiments suggesting that the actin-based mechanism of force generation that drives the persistent locomotion of *Dictyostelium* cells in narrow channels may be similar to the mechanism underlying interstitial neutrophil migration [17]. In particular, we observed that a dense adherent actin network grows inwards from the contact regions with the side walls, while a highly dynamic, loose network dominates the leading edge. Myosin II activity is not required in this process. In contrast to the earlier results from neutrophils, where directional movement was induced by chemoattractant gradients, no chemoattractants were present in our experimental setup. Instead, we observed that amoeboid cells spontaneously entered this state of mechanical polarity under the influence of confinement that is characterized by a persistent, unidirectional motion along the microchannel.

### A model for confinement-induced polarity

How can we explain the geometry-induced mechanical polarity in motile amoeboid cells? We will introduce a phenomenological reaction-diffusion model to search for mechanisms that may explain our observations. In this framework, the intracellular cytoskeletal processes are described based on a small number of effective components that can spread diffusively throughout the cell and interact according to given kinetic relations. These components do not directly correspond to molecular players in the cell but they are rather chosen such that they recover the main dynamical features of the cytoskeletal machinery. Based on a model of this type, we may test basic properties and overall trends like dependencies on the cell size or qualitative changes in the dynamical behavior as a function of changes in external parameters.

**Figure 5:**
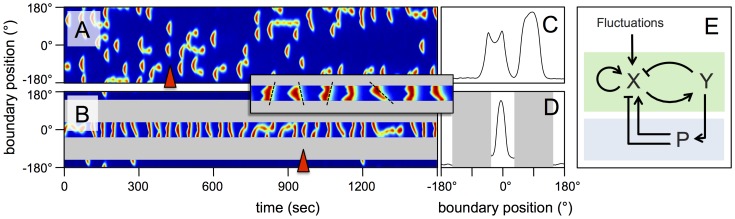
An excitable network model accounts for confinement-induced polarity. (A) Kymograph of model variable showing the formation of patches that represent randomly emerging pseudopods. (B) Kymograph of model variable in the presence of channel boundaries (gray). All model parameters are chosen identically in (A) and (B). Inset: Zoom of structures forming between 400 and 700 sec. Examples of slanted structures are indicated by dashed black lines to guide the eye. (C,D) Spatial profiles of at the time points indicated by red wedges in (A) and (B), respectively. (E) Schematic diagram of the excitable network model (green) with polarity module (blue).

Several models of this type have been proposed that describe actin-based protrusion dynamics at the leading edge based on excitable reaction-diffusion systems [29]–[32], for a review see also [33]. Here, we focus on the biased excitable network model that relies on a generic, excitable FitzHugh-Nagumo-type system, which is composed of an autocatalytic component *X* that drives its own inhibitor *Y* [29]. We consider a one-dimensional system with periodic boundary condition, where the spatial distribution of the two species reflects the protrusive activity along the cell membrane. Due to an additional noise term, excitations are triggered from time to time at different locations of the system, representing the spontaneous formation of pseudopods in a freely moving cell. For a schematic representation of the dynamics of this system, see the network diagram in the green shaded part of Figure 5E. In the following, we will modify and extend this model to take the presence of the channel boundaries into account. It is the aim of this approach to provide an explanation for the polarized state of the persistent walkers that we observed in our experiments. In particular, we want to elucidate, why pseudopods that are respresented by randomly emerging, localized excitations in the model, are formed only at one side of the cell when confined in a narrow channel. We consider the biased excitable network model an ideal framework to explore this question because it represents a minimal mathematical model that qualitatively captures the dynamics of pseudopod formation and offers straightforward ways to include polarization.

In the originally proposed biased excitable network model, the excitable system was coupled to a local-excitation, global-inhibition (LEGI) module, where a slowly diffusing local activator and a fast diffusing inhibitor are released upon a receptor stimulus, so that cytoskeletal activity is initiated preferentially at those parts of the membrane, where the activator concentration exceeds the inhibitor [34], [35]. Different variants of this model have been proposed [36]–[38]. The LEGI system imposes a spatial bias on the excitability to account for directional pseudopod formation in response to an external chemoattractant gradient [29], [30]. In subsequent work, cell polarity, and the mechanics of the cell cortex were included in the same type of model to account for the full cellular morphology during cell motion [39]. Recent results demonstrate that the cytoskeletal machinery may also show oscillatory behavior [22], [40], [41], a finding that has also been incorporated into the approach of excitable network modeling [41]. Here, we use a reduced version of the biased excitable network model, including only the excitable component that accounts for pseudopod formation (green shaded part in Figure 5E) together with a polarity module (the blue shaded part). We incorporate polarity via a single LEGI-type variable such that the probability for pseudopod formation is increased in regions where previous pseudopods were located. In all other regions, the probability for pseudopod formation is reduced. The variable encodes the degree of polarity and is linked to the cytoskeleton through a positive feedback loop, a common feature of many polarity models [42] that is supported by experimental findings in both neutrophils and *Dictyostelium* [43], [44].

The model is built to account for the dynamics of protrusion formation at the open membrane of an amoeboid cell. However, in regions where the cell is in direct contact with the microchannel side walls, pseudopod formation is prohibited due to the balancing forces exerted by the channel walls. These regions are therefore not part of the dynamical system that is described by our model. We incorporate the contact areas into the model as gaps in the computational domain that divide the system into two smaller subdomains of ‘active’ membrane area (cell front and back). For details of the model equations and parameters refer to the Supporting Information.

We performed numerical simulations to account for our experimental setting. Parameters were chosen such that the cell exhibits moderate polarity in agreement with the developmental state of the cells in our experiments, i.e., the probability of pseudopod formation was slightly increased in regions where a pseudopod has been formed previously, corresponding to a non-zero polarity parameter (, see also the model equations in the Supporting Information). In Figure 5A, a kymograph of the corresponding numerical simuation is shown. It represents a freely moving cell in absence of any confinement, with pseudopods randomly distributed across the cell membrane. A typical profile of the model variable along the cell perimeter is displayed in Figure 5C, corresponding to the point in time indicated by a red wedge in Figure 5A. In contrast, if cells are confined between the two side walls of the microchannel, a highly asymmetric pattern emerges for the same set of parameters. Pseudopods form almost exclusively on one side of the cell (front), while the other side (back) remains quiescent (Figure 5B). This numerical result corresponds to the persistent, unidirectional motion of the persistent walkers observed in our experiments (cf. Figure 2E). A *Y*-profile along the cell perimeter corresponding to the time point indicated in Figure 5B (red wedge) is shown in Figure 5D.

A closer comparison reveals qualitative agreement between the experimental data in Figure 2E and the numerical results in Figure 5B. In both cases, protrusions are formed periodically, between one and two per minute. Most likely, the uniform movement of the persistent walkers is a consequence of this highly regular, periodic protrusion pattern. The protrusions travel across the front membrane in a wave-like fashion, leading to slanted structures. This pattern is consistently observed in both the experimental and numerical kymographs. Their speed is similiar to the speed of curvature waves that have been observed for cells migrating on open planar substrates [22]. We also note that the left/right alternating protrusion of the cell front can be seen as a confined version of the zigzag pattern of pseudopod extension that has been observed for cells moving on flat surfaces [45], [46]. In the model, the alternating protrusion pattern is most likely related to the characteristic size of the self-organized structures displayed in Figure 5A and to the fact that new structures tend to emerge at the corner positions, where previous structures died out. In a channel with a width similar to the size of these structures, a randomly emerging structure is most likely not initiated exactly in the middle of the channel, so that one side of it will be extinguished at the wall while the other side travels further across the channel and marks the position where a new structure will be born later. In this way, a zigzag pattern may emerge.

The model suggests that polarity is increased if the active membrane area, where pseudopods can be formed, is reduced. This is also confirmed by numerical simulations with other geometrical constraints of the computational domain (see Figure S4). For a smaller active membrane area, the concentration of signaling components associated with cell polarization is increased, which results in a higher degree of polarity. This is furthermore supported by the fact that we do not find any persistent walkers in microchannels that are 20 µm in width. In these channels, cells are not able to stay in touch with both side walls at the same time. Our model suggests that they are less polarized because, with contact to only one side wall, they maintain a larger active membrane area. In narrower microchannels (5 µm in width), we did not observe any persistent walkers either. This is in agreement with earlier observations of optimal confinement for T cell migration, where mechanical friction impairs efficient migration in channels much narrower than the diameter of the cells [27]. We believe that similar effects can explain why we do not observe persistent motion in channels much narrower than 10 µm. However, the current version of our model does not include mechanical friction and thus does not recover this observation.

Also our finding that myosin II is not essential for persistent motion under confinement is in line with our model. The degree of confinement-induced polarity depends on the active membrane area, where pseudopods can form and does not rely on contractile forces at the rear of the cell. To further test the validity of our model, we also analyze collision scenarios between persistent walkers and other cells inside the microchannel. Upon a collision, the persistent walker may either traverse the region of the other cell (above or below the other cell) and continue moving in its original direction, or it may collide with the cell and, upon collision, reverse its direction of motion. Our model successfully captures such collision events. The corresponding experimental data together with the numerical results are presented in Figure S3.

While we explain our finding based on a phenomenological model of the cytoskeletal activity, other descriptions have been proposed to account for persistent motion in confined geometries. In the case of epithelial cells, self-generated gradients in epidermal growth factor are a likely reason for directed migration [14]. Note, however, that in the data presented here, cells do not occlude the entire channel, so that the buildup of self-generated gradients across the cells is very unlikely. Similarly, an influence of hydraulic pressure or water permeation effects can be excluded in our case [19], [20]. Also a more generic scheme based on velocity alignment of active Brownian particles has been recently proposed to account for persistent motion of confined cells [47].

## Conclusion

In summary, our experimental findings show that motile amoeboid cells can undergo a spontaneous symmetry breaking under the influence of mechanical confinement. They exhibit highly persistent, unidirectional motion associated with a characteristic arrangement of the actin cytoskeleton. Dense actin-rich regions are observed in contact areas with the side walls and a less dense dynamic region of polymerizing actin mediates the formation of protrusions at the leading edge. An excitable network model accounts for this confinement-induced polarity and for the observed periodic formation of protrusions that travel across the front membrane in a wave-like fashion.

Since the persistent motion is associated with well-defined intracellular dynamics, our results open up new possibilities to investigate the signaling pathways and feedback loops that are triggered by mechanical stimulation of cells in detail. In future experiments, we will use this setting together with different fluorescently labeled *Dictyostelium* cell lines to probe the subcellular localization of signaling components in cells that exhibit mechanically induced polarity. Furthermore, we note that the natural habitat of many motile cells is typically dominated by surfaces, boundaries, and narrow interstitial spaces. This holds for the surrounding tissue of leukocytes or metastatic cancer cells as well as for the granular soil environment of *Dictyostelium* amoeba. We expect that geometry-induced polarity may be ubiquitous in such surroundings and could assist cells in navigating efficiently through complex porous environments.

## Supporting Information

Figure S1 **Analysis of cortical dynamics at the wall-attached cell membrane.** (A) Definition of the wall-attached zone and the front and back corners. (B) Actin intensity profile in the wall-attached cortex. The inset demonstrates how these profiles are stacked to a kymograph. The tilted shape of the kymograph results from the persistent unidirectional motion of the cell along the microchannel.
(TIFF)

Figure S2 **Actin localization in a myosin II-null persistent walker.** (A) Kymograph of the LimE-GFP distribution in a persistently moving myosin II-null cell. (B) Snapshot of a LimE-GFP expressing myosin II-null cell during persistent motion.
(TIFF)

Figure S3 **Collision of a persistent walker with a random walker.** (A) Snapshots before (0 min), during (8 min), and after the collision (12 min). The persistent walker is entering from the left and, after the collision, leaving to the left, see also Movie 3. Cell positions are marked by white wedges. (B) Kymograph of the local motion. Protruding regions appear in red and retracting regions in blue. The walls, where neither protrusion nor retraction takes place, are shown in green/light blue. Upon collision at around 500 sec, the cell reverses direction, clearly indicated by a switch in the position of the protrusive activity from one side of the cell to the other. (C) Kymograph of a corresponding model simulation. The collision is incorporated into the model by shutting off the subdomain, where persistent pseudopod formation occurred, for a short interval of time (corresponding to the collision time), so that during collision the formation of further pseudopods is prohibited at the cell front. Pseudopods then emerge on the remaining subdomain at the back of the cell and thus could induce a switch in the direction of polarity.
(TIFF)

Figure S4 **Cell polarity depends on system size.** Upon a decrease of the system size from (A) 600 over (B) 400 to (C) 200 space units, a clear increase in polarity is observed. While pseudopods are randomly distributed in the case of , new pseudopods exclusively form in places, where previous pseudopods have been located in the case of . Parameters were chosen as listed in the Appendix except for , which was increased to to yield a more pronounced effect for reasons of illustration.
(TIFF)

Figure S5 **Layout of the microfluidic device.** (Top) Entire microfluidic structure with inlet and outlet regions. (Bottom) Zoom of the center region with narrow microchannels. A cascade of channels with 20 µm (left), 10 µm (middle), and 5 µm width can be seen that are connected by wide rectangular reservoir regions.
(TIFF)

Figure S6 **Correlations of the front corners of persistent walkers.** (A) Biased autocovariance functions from the front corners of one cell (gray: left corner, black: right corner). Blue and red dots mark local extrema, used to measure the periodicity of the oscillations. (B) Biased crosscovariance function between the left and right front corners. Blue and red dots show local extrema. (C) Box plots of timescales obtained from 9 cells. (i) Average periodicity of autocorrelations of left and right front corners: min. (ii) Average periodicity of the cross correlation: min. (iii) Average delay between left and right front corner oscillations: sec. Error bars display the standard error of the mean.
(TIFF)

Movie S1 **Examples of persistent walkers.** Between 15 and 40 min a persistent walker can be seen that is only in contact with one sidewall. Between 45 and 74 min the persistent walker corresponding to Fig. 1B can be seen.
(MOV)

Movie S2 **Example where cells on an open surface and under confinement can be observed at the same time.** Cells on the open surface are clearly not polarized. Persistent walkers can be seen, e.g. between 0 and 14 min in the upper channel (from right to left), between 30 and 47 min in the upper channel (from left to right), and between 75 and 100 min in the bottom channel (from left to right).
(MOV)

Movie S3 **Colliding cells (bottom channel).** First, a persistent walker, coming from the left, passes across the random walker. At 68 min, a second persistent walker enters from the left and is reflected upon collision with the random walker, corresponding to the snapshots shown in Fig. S3A.
(MOV)

Movie S4 **Actin dynamics imaged via expression of LimE-RFP in a persistent walker.**
(MOV)

Movie S5 **Myosin II-GFP dynamics in a persistent walker (same cell as in Movie 4).**
(MOV)

Movie S6 **Actin dynamics imaged via expression of LimE-GFP in a persistently walking myosin II-null cell.**
(MOV)

Movie S7 **Three-dimensional reconstruction from a spinning disk microscopy recording of a persistent walker expressing LimE-RFP and myosin II-GFP.**
(MOV)

Appendix S1 **Image analysis and modeling.** Cell shape and motion analysis, detection and analysis of the cell boundary regions that are in contact with the microchannel wall, measuring the life time of actin foci, model equations and parameters.
(PDF)
