# Supplementary figures and images for: Geometry-Driven Polarity in Motile Amoeboid Cells
Source: PLoS One. 2014 Dec 10;9(12):e113382. doi: 10.1371/journal.pone.0113382 (PMC4262208; doi:10.1371/journal.pone.0113382)

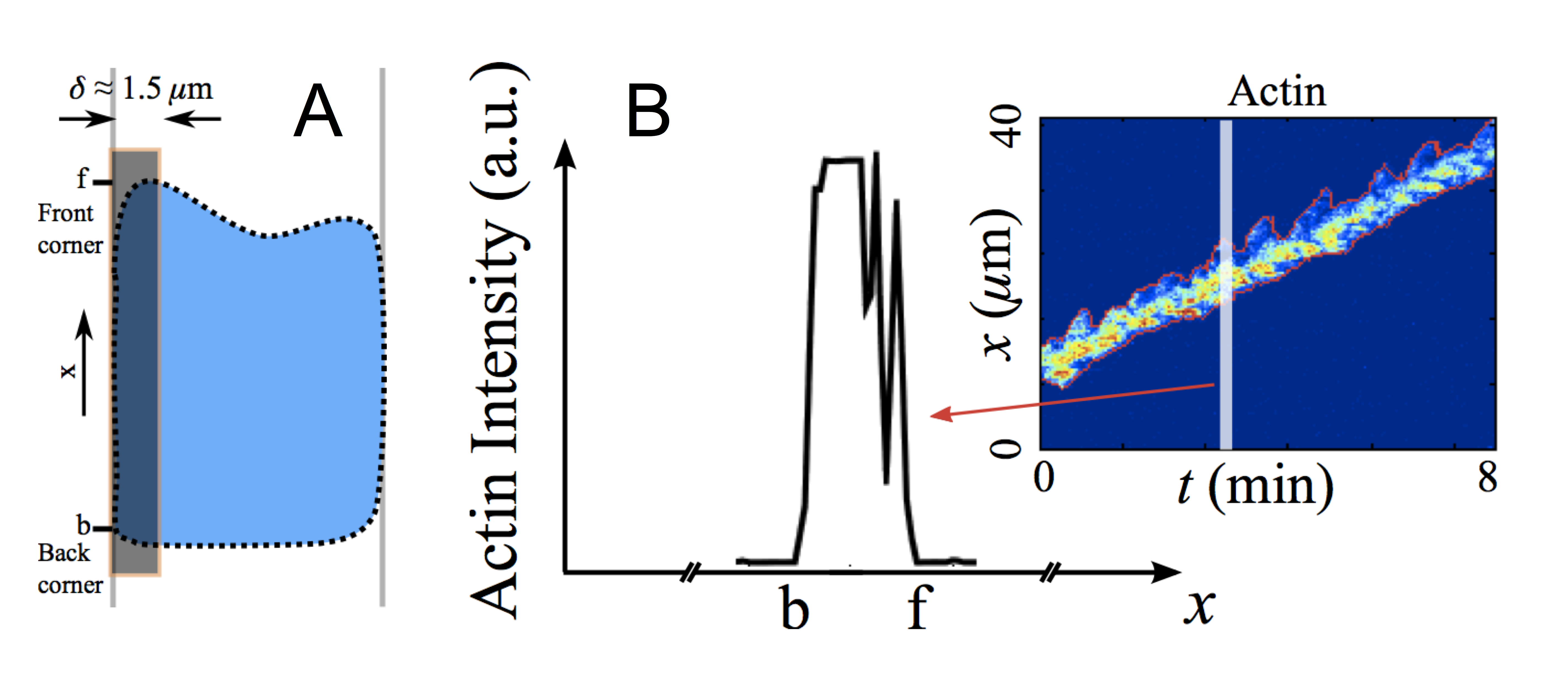

Supplement: Figure S1 — Analysis of cortical dynamics at the wall-attached cell membrane. (A) Definition of the wall-attached zone and the front and back corners. (B) Actin intensity profile in the wall-attached cortex. The inset demonstrates how these profiles are stacked to a kymograph. The tilted shape of the kymograph results from the persistent unidirectional motion of the cell along the microchannel. (TIFF) [file pone.0113382.s001.tiff]

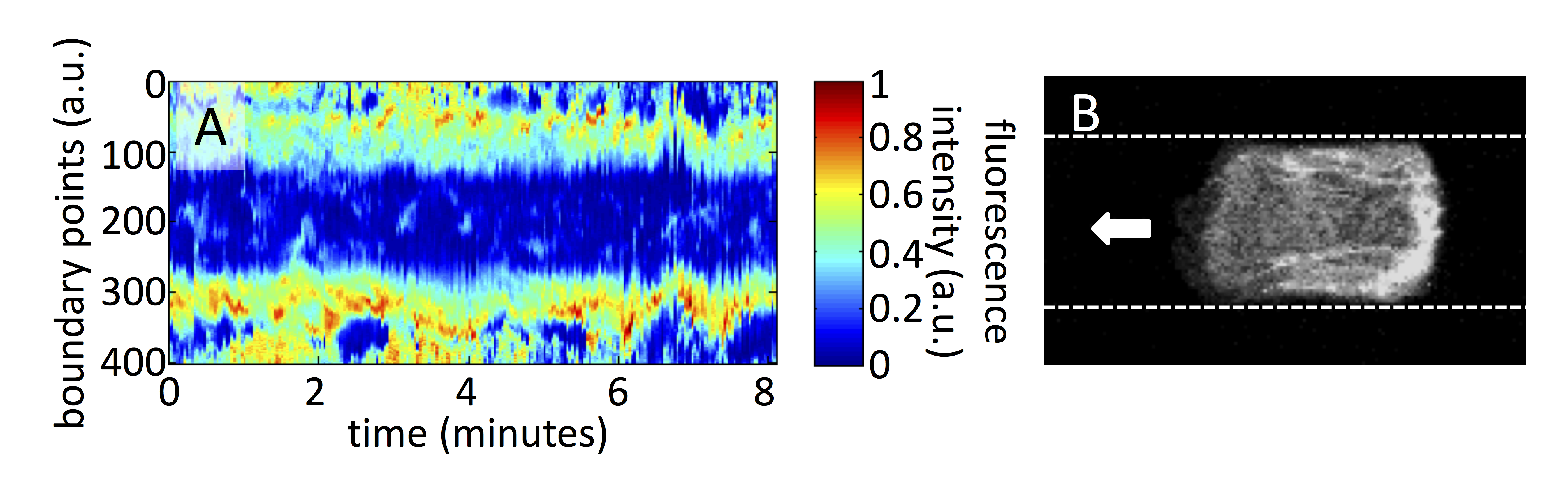

Supplement: Figure S2 — Actin localization in a myosin II-null persistent walker. (A) Kymograph of the LimE-GFP distribution in a persistently moving myosin II-null cell. (B) Snapshot of a LimE-GFP expressing myosin II-null cell during persistent motion. (TIFF) [file pone.0113382.s002.tiff]

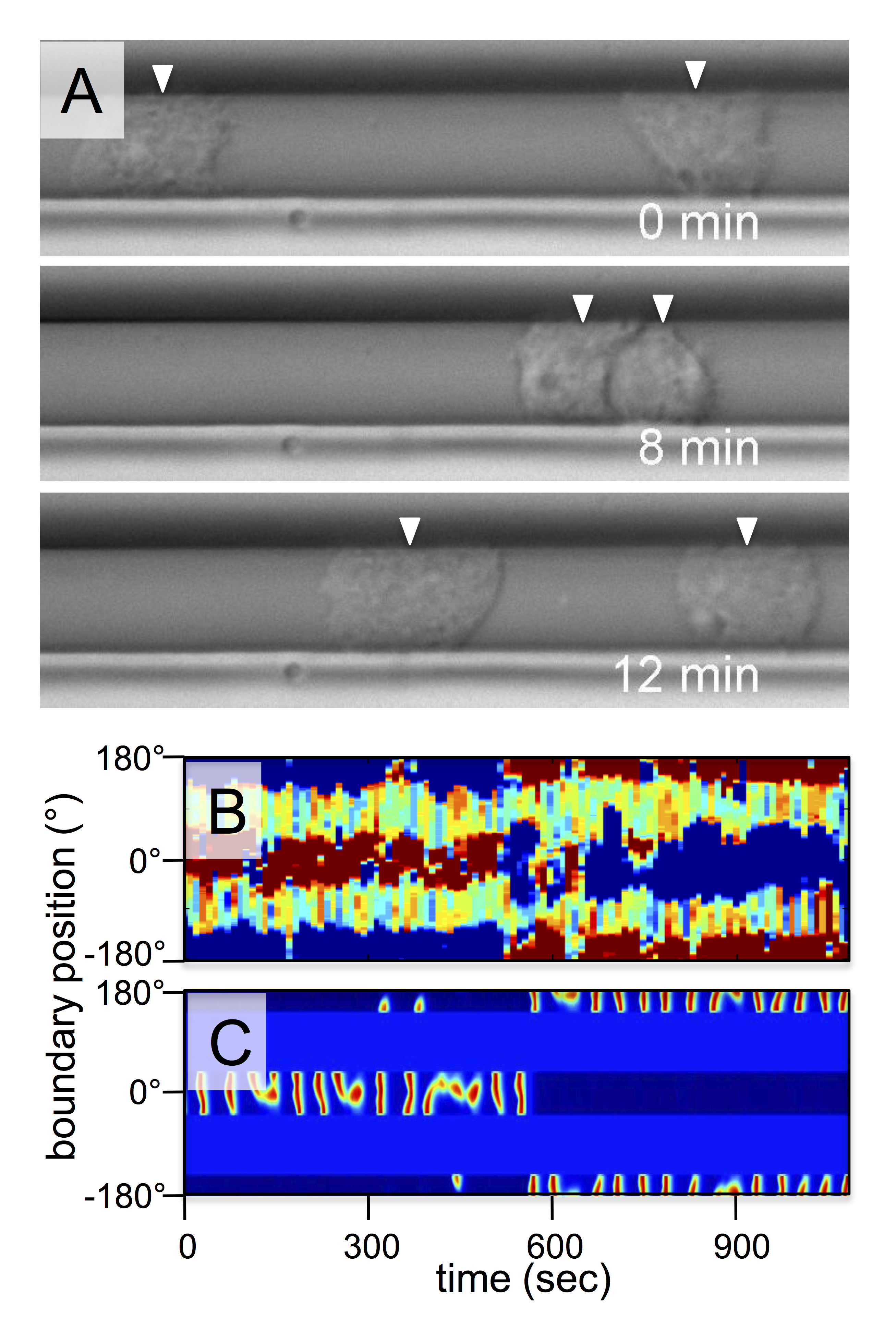

Supplement: Figure S3 — Collision of a persistent walker with a random walker. (A) Snapshots before (0 min), during (8 min), and after the collision (12 min). The persistent walker is entering from the left and, after the collision, leaving to the left, see also Movie 3. Cell positions are marked by white wedges. (B) Kymograph of the local motion. Protruding regions appear in red and retracting regions in blue. The walls, where neither protrusion nor retraction takes place, are shown in green/light blue. Upon collision at around 500 sec, the cell reverses direction, clearly indicated by a switch in the position of the protrusive activity from one side of the cell to the other. (C) Kymograph of a corresponding model simulation. The collision is incorporated into the model by shutting off the subdomain, where persistent pseudopod formation occurred, for a short interval of time (corresponding to the collision time), so that during collision the formation of further pseudopods is prohibited at the cell front. Pseudopods then emerge on the remaining subdomain at the back of the cell and thus could induce a switch in the direction of polarity. (TIFF) [file pone.0113382.s003.tiff]

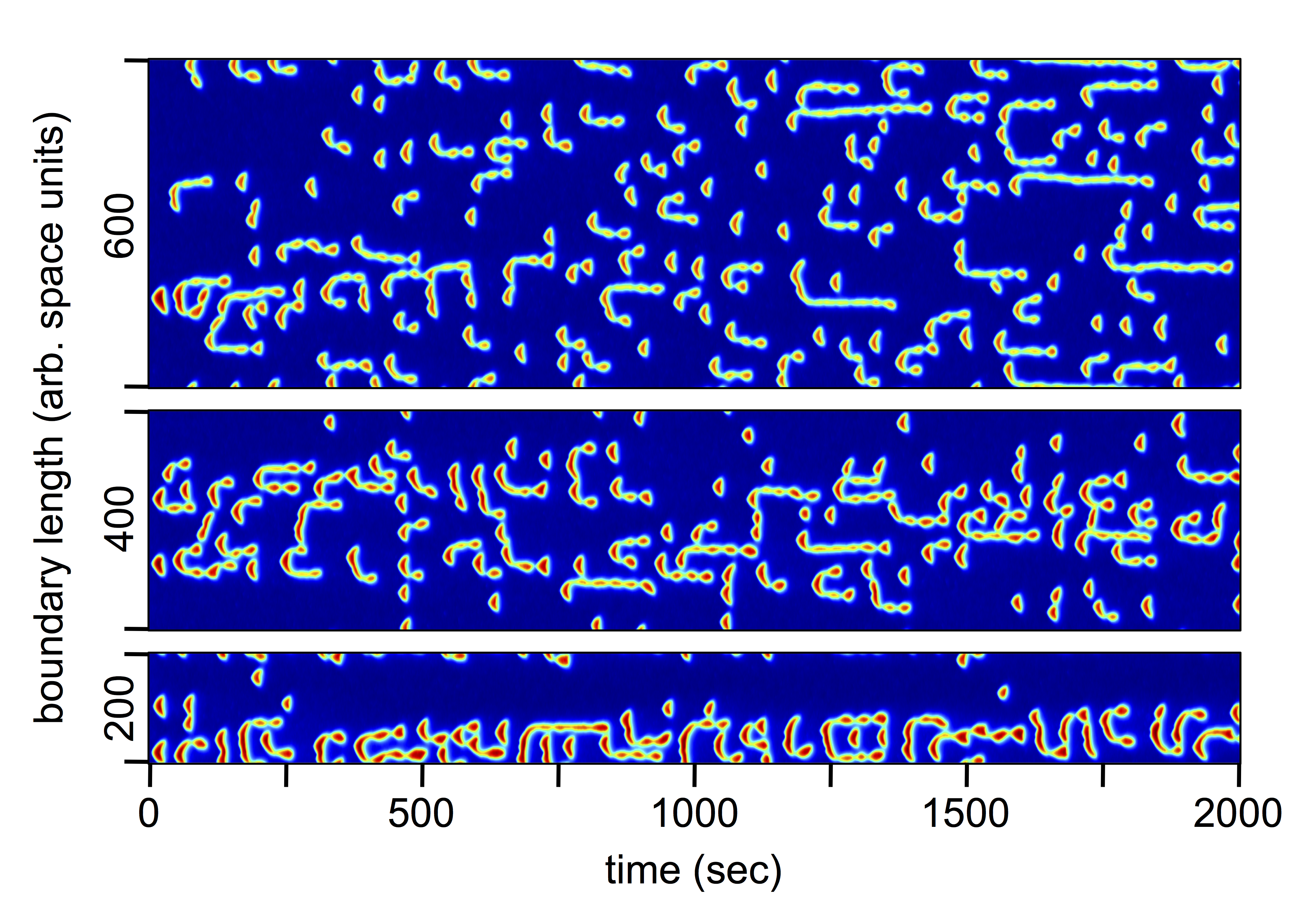

Supplement: Figure S4 — Cell polarity depends on system size. Upon a decrease of the system size from (A) 600 over (B) 400 to (C) 200 space units, a clear increase in polarity is observed. While pseudopods are randomly distributed in the case of , new pseudopods exclusively form in places, where previous pseudopods have been located in the case of . Parameters were chosen as listed in the Appendix except for , which was increased to to yield a more pronounced effect for reasons of illustration. (TIFF) [file pone.0113382.s004.tiff]

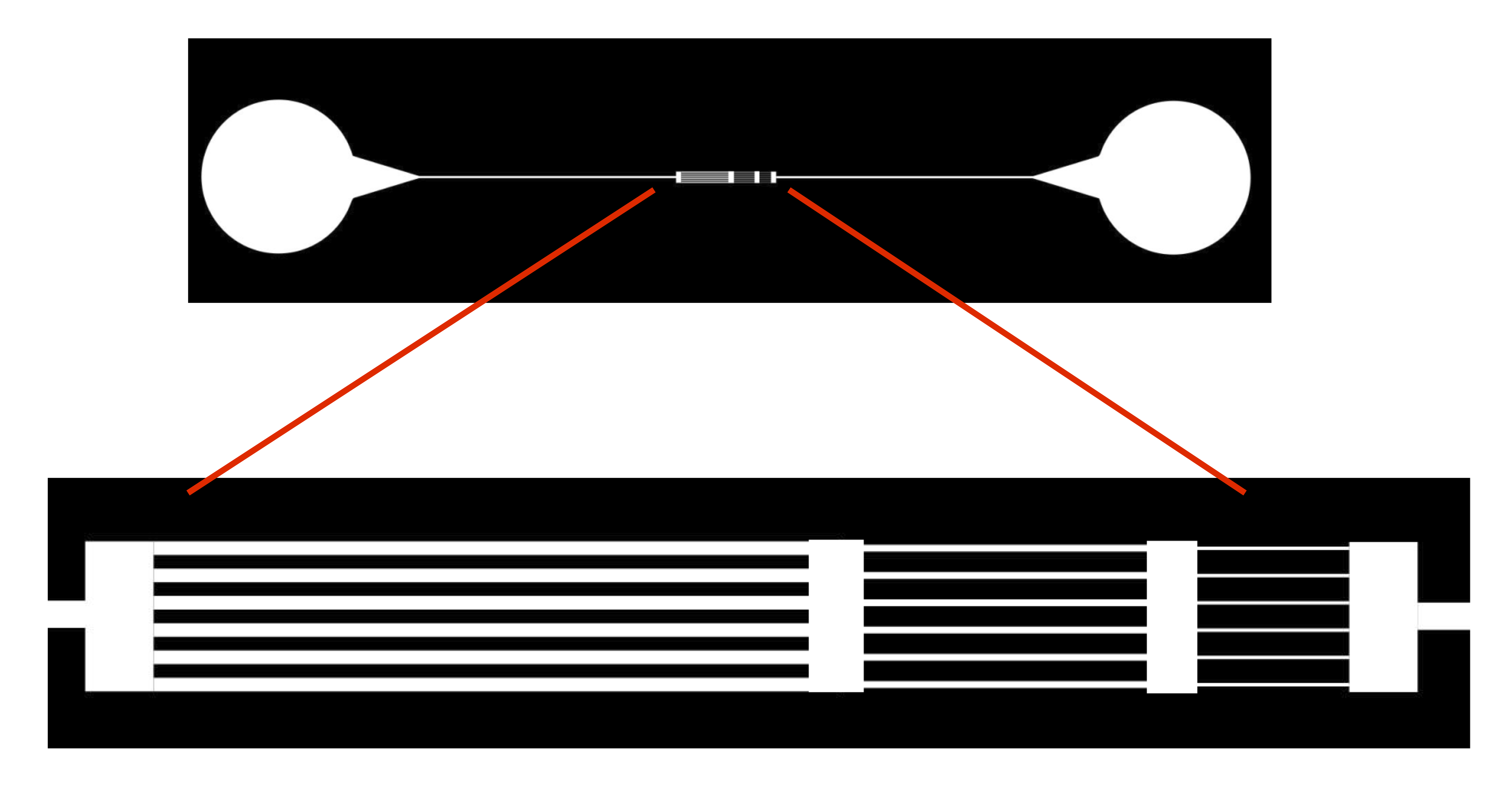

Supplement: Figure S5 — Layout of the microfluidic device. (Top) Entire microfluidic structure with inlet and outlet regions. (Bottom) Zoom of the center region with narrow microchannels. A cascade of channels with 20 µm (left), 10 µm (middle), and 5 µm width can be seen that are connected by wide rectangular reservoir regions. (TIFF) [file pone.0113382.s005.tiff]

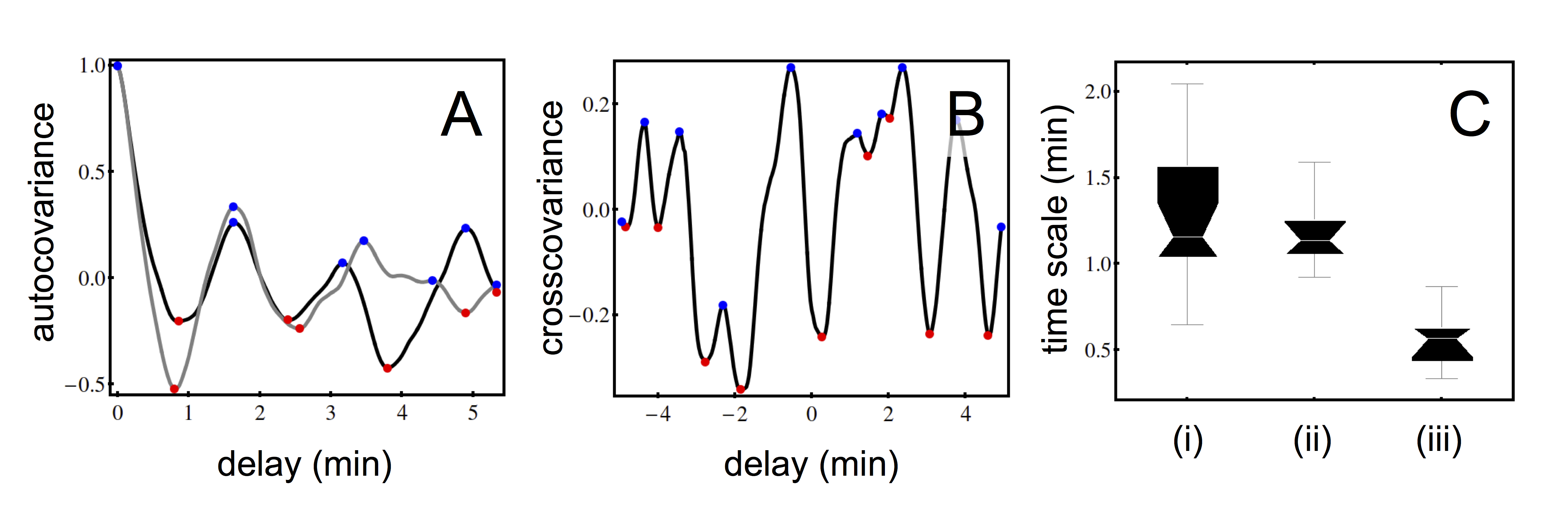

Supplement: Figure S6 — Correlations of the front corners of persistent walkers. (A) Biased autocovariance functions from the front corners of one cell (gray: left corner, black: right corner). Blue and red dots mark local extrema, used to measure the periodicity of the oscillations. (B) Biased crosscovariance function between the left and right front corners. Blue and red dots show local extrema. (C) Box plots of timescales obtained from 9 cells. (i) Average periodicity of autocorrelations of left and right front corners: min. (ii) Average periodicity of the cross correlation: min. (iii) Average delay between left and right front corner oscillations: sec. Error bars display the standard error of the mean. (TIFF) [file pone.0113382.s006.tiff]
