# Supplementary material for: Geometry-Driven Polarity in Motile Amoeboid Cells
Source: PLoS One. 2014 Dec 10;9(12):e113382. doi: 10.1371/journal.pone.0113382 (PMC4262208; doi:10.1371/journal.pone.0113382)
Supplement: Appendix S1 — Image analysis and modeling. Cell shape and motion analysis, detection and analysis of the cell boundary regions that are in contact with the microchannel wall, measuring the life time of actin foci, model equations and parameters. (PDF) [file pone.0113382.s014.pdf]

Appendix

Cell shape and motion analysis

We use an active contour algorithm to extract the shapes of the *D. discoideum* cells [1]. The cell boundary, which is described by 400 boundary points, is locally tracked from frame to frame using a least square mapping. Furthermore the centroid of the cell is computed. For each frame the distance of the boundary points to the centroid is calculated, resulting in a quantitative measure of the cell shape. The algorithm also displays the local motion of the boundary points by comparing changes in the boundary point positions from one frame to the next. Finally, we measure the concentrations of F-actin and myosin II at each boundary point by calculating the total intensities of the associated fluorescent markers in a small region around each boundary point. We thus obtain the spatio-temporal distributions of F-actin and myosin II along the cell border.

Detection and analysis of the cell boundary regions that are in contact with the microchannel wall

To find the cell boundary regions that are in contact with the microchannel walls, we use a gradient-based edge detection algorithm. We first find the position of the microchannel walls from the summed fluorescence intensities of the actin and myosin II markers. We average the summed intensity with respect to time and position x (the direction parallel to the motion of the cell). The time average gives the average contact region of the cell with the microchannel wall, and the x-average minimizes the noise. This averaging yields a typical intensity profile that is narrow and rapidly decreasing close to the microchannel wall. We find pixels that are close to the microchannel wall by selecting those which have an intensity below 1% of the total intensity. Next, we calculate the derivative of the intensity for these points. Then we look for a sudden change in the derivative by performing a unidirectional search that starts from outside the microchannel wall and scans towards the cell cytoplasm. The sudden change in the derivative ranges from 0.0005 to 0.005. We choose a different value for each cell, because the fluorescence intensity of the labeled proteins depends on the expression level and varies from cell to cell.

Next, we find the front-most and back-most boundary points, where the cell is in contact with the microchannel wall, see Fig. S1A. At each x-position, we sum the total intensity starting from the microchannel wall over a distance of 6 pixels ($\approx 1.44\ \mu$m) into the cytoplasm. An example of the resulting intensity profile along the channel wall is shown in Fig. S1B. We next employ a similar unidirectional search as above for the locations of the anterior and posterior contact. We search for 5 consecutive points that each have an intensity above a threshold value. For each cell, we individually choose a threshold intensity in the range of $[0.002, 0.005]$. The time evolution of the front-most and back-most locations of the cell contact with the microchannel are shown as red outlines in the inset in Fig. S1B.

Measuring the life time of actin foci

To obtain the life time of the actin foci on the cell membrane in contact with the microchannel wall (at a given boundary position), we consider actin intensities that are greater than $\epsilon = 78\%$ of the maximum intensity (thresholding). Next, we calculate the average and the standard deviation of the intensity in the thresholded region over time. We then smooth the actin intensity using "lowess" method in MATLAB's smooth function. We monitor the deviation of this smoothed intensity at each time point in the region from the time-averaged intensity and count the number of frames during which the deviation is within $\Delta = 2\%$ of the average intensity. The values of ϵ and Δ are picked such that the distribution obtained from one kymograph has maximum similarity with the one that is manually obtained ($p \approx 0.85$ using Kolmogorov-Smirnov test). For the remaining kymographs, we utilized the algorithm to obtain the distribution of actin foci life time shown in Fig. 4C ($n = 10$ cells).

Model equations and parameters

To interpret our experimental findings, we performed numerical simulations of an excitable network model with an additional polarity module. We used a reduced version of the model that was introduced by Xiong et al. [2]. As there were no chemotactic signals present in our experiments, we based our description solely on the excitable part of the model by Xiong et al. and omitted the LEGI module that was introduced to account for receptor input signals. In this model, excitable dynamics is described by a FitzHugh-Nagumo-type two variable system, where the autocatalytic activator X stimulates production of the inhibitor Y that downregulates production of X,

$$\frac{\partial X}{\partial t} = \alpha\left[\frac{(a+1)X^{2n}}{a+X^{2n}} - X - \beta Y\right] + U + D_X \nabla^2 X\,, \qquad \text{(S1)}$$

$$\frac{\partial Y}{\partial t} = \alpha\epsilon(\gamma X - Y) + D_Y \nabla^2 Y\,. \qquad \text{(S2)}$$

Both the activator and the inhibitor can diffuse with D_X and D_Y denoting the respective diffusion coefficients. The values of the coefficients a, α, β, γ, and ϵ are given in the Table below. The Hill coefficient $2n$ controls the nonlinearity in the autocatalytic reaction and U sets the excitability threshold of the system. For a more detailed phase plane and bifurcation analysis see the Supporting Information of Ref. [2].

In a recent publication, Shi et al. have extended this model to take cell polarity into account [3]. They achieve this by including an additional two-variable LEGI module consisting of a locally activating species and a second component that exerts a global negative feedback. Here, we mimic this effect by a single cortical polarity variable P. Recruitment of P to the cortex depends on the amount of locally available Y,

$$\frac{\partial P}{\partial t} = k_1 Y P_{\mathrm{pool}} - k_2 P + D_P \nabla^2 P \,, \tag{S3}$$

with k_1 and k_2 the on and off rates for the recruitment of P to the cell cortex and D_P the diffusion coefficient of P. The pool P_{pool} of available non-bound P is given by

$$P_{\mathrm{pool}} = P_{\mathrm{tot}} - \int_L P \,\mathrm{d}x \,, \tag{S4}$$

with P_{tot} the total amount of P in the system and L the system size. The polarity variable P couples back to the excitable system by locally influencing the excitability threshold according to

$$U = \theta + \phi \left(P - \frac{1}{L} \int_L P \,\mathrm{d}x \right) . \tag{S5}$$

Here, the average value of P across the cell corresponds to the second globally inhibiting polarity variable in the model by Shi et al. [3]. The parameters θ and ϕ are given in the Table below. The system size L denotes the active membrane area, where pseudopods can form. In the presence of walls, L is reduced to the total system size minus the extent of the walls.

In our simulations, we add noise to the activator variable X in such a way that excitations are randomly triggered to represent pseudopod formation. Similar to the approach of Xiong et al., we introduce the noise as a Wiener process W so that Eq. (S1) becomes a stochastic differential equation,

$$\mathrm{d}X_t = \left(\alpha \left[\frac{(a+1)X_t^{2n}}{a + X_t^{2n}} - X_t - \beta Y_t \right] + U_t + D_X \nabla^2 X_t \right) \mathrm{d}t + \sigma \mathrm{d}W_t \,. \tag{S6}$$

The noise term is discretized with time step $\mathrm{d}t$ according to $\mathrm{d}W \sim \sqrt{\mathrm{d}t}\, N(0,1)$.

The channel walls are included as gaps in the computational domain, and we impose no-flux boundary conditions at the positions where the active part of the membrane ends.

Table 1. Model parameters. All numbers are taken from [2], except the parameters for the polarity module.

Parameter	Value
a	0.1
n	1
α	2.870
β	3.711
γ	0.214
ϵ	0.038
σ	0.102
θ	-0.035
ϕ	5
k_1	0.1
k_2	0.01
P_{tot}	1
D_X	1.864
D_Y	4.773
D_P	2

References

1. Driscoll MK, McCann C, Kopace R, Homan T, Fourkas JT, et al. (2012) Cell shape dynamics: From waves to migration. PLoS Comput Biol 8: e1002392.

2. Xiong Y, Huang CH, Iglesias PA, Devreotes PN (2010) Cells navigate with a local-excitation, global-inhibition-biased excitable network. PNAS 107: 17079–17086.

3. Shi C, Huang CH, Devreotes PN, Iglesias PA (2013) Interaction of motility, directional sensing, and polarity modules recreates the behaviors of chemotaxing cells. PLoS Comput Biol 9: e1003122.
